# Interplay between Vitamin D and Sphingolipids in Cardiometabolic Diseases

**DOI:** 10.3390/ijms242317123

**Published:** 2023-12-04

**Authors:** Simona Fenizia, Melania Gaggini, Cristina Vassalle

**Affiliations:** 1 Department of Sciences and Technological Innovation, University of Piemonte Orientale, Corso Trieste 15/A, I-28100 Novara, Italy; simona.fenizia@gmail.com; 2 Department of Translational Medicine, University of Piemonte Orientale, Corso Trieste 15/A, I-28100 Novara, Italy; 3 Istituto di Fisiologia Clinica, Italian National Research Council, Via Moruzzi 1, I-56124 Pisa, Italy; melania.gaggini@ifc.cnr.it; 4 Fondazione CNR-Regione Toscana G. Monasterio, Via Moruzzi 1, I-56124 Pisa, Italy

**Keywords:** 1,25-dihydroxyvitamin D, sphingolipids, ceramides, sphingosine 1-phosphate, vitamin D, calcitriol, crosstalk, cardiometabolic risk, cardiometabolic disease

## Abstract

Sphingolipids (SLs) are structural, bioactive molecules with several key cellular roles, whereas 1,25-dihydroxyvitamin D (1,25(OH)D), the active form of vitamin D, is considered the major regulator of calcium homeostasis, although it also exerts other extraskeletal effects. Many studies reported the physiological connection between vitamin D and SLs, highlighting not only the effects of vitamin D on SL metabolism and signaling but also the influence of SLs on vitamin D levels and function, thus strongly suggesting a crosstalk between these molecules. After a brief description of 1,25(OH)D and SL metabolism, this review aims to discuss the preclinical and clinical evidence on the crosstalk between SLs and 1,25(OH)D, with a special focus on cardiometabolic diseases.

## 1. Introduction

Vitamin D is a well-known key factor in bone health, as its active form, 1,25-dihydroxyvitamin D (calcitriol, 1,25(OH)D), affects intestinal absorption of calcium and phosphate, both of which are essential elements for proper bone mineralization [1]. However, growing evidence suggests that vitamin D deficiency may play a pathogenic role in other diseases (i.e., cardiometabolic, neurodegenerative, and cancer) since many cell types in different organs harbor vitamin D receptors (VDRs) and respond to vitamin D, which thus modulates the expression of genes involved in many critical cellular functions [2,3,4]. In particular, these genomic effects are mediated by the nuclear translocation of the 1,25(OH)D-cytosolic VDR complex, as well as by binding with the 9-cis-retinoic acid receptor (RXR), followed by further binding to vitamin D response elements (VDREs) on DNA, resulting in the modulation (inhibition or enhancement) of many protein synthesis pathways [5].

However, some effects are too fast to be attributed to changes in gene expression but rather are to be linked to the membrane-VDR (mVDR) and appear to involve MARRS (membrane-associated, rapid response steroid-binding protein), thus affecting several signal transduction pathways [6]. Noteworthy is the role of vitamin D in metabolic alterations (e.g., obesity and type 2 diabetes), which are highly involved in the onset and development of cardiovascular (CV) disease; indeed, low vitamin D levels have been associated with type 2 diabetes and obesity, whereas vitamin D supplementation may improve glycemic control in type 2 diabetes patients, as well as insulin secretion and sensitivity [7,8]. Additionally, results obtained from several studies highlight not only the association between nonalcoholic fatty liver disease (NAFLD), the most common cause of chronic liver disease, and vitamin D deficiency but also suggest that vitamin D supplementation may be beneficial in NAFLD (e.g., improving intestinal permeability, as well as intestinal microbial metabolites, hepatic inflammation, and liver fibrosis) [9,10].

Moreover, different studies demonstrated a significant association between low levels of circulating 25(OH)D and an increased prevalence and extent of ischemic heart disease, as well as risk of CV events, including mortality [11]. In particular, among acute myocardial infarction (AMI) patients, there is a very high percentage of individuals with hypovitaminosis D; hence, in a study of an Italian AMI cohort, we showed a high prevalence of severe vitamin D deficiency, particularly in female patients, which may explain (almost in part) the sex differences in the pathophysiology of AMI [4,12].

Based on epidemiological and preclinical studies, there is further evidence of an association between hypovitaminosis D and cerebrovascular diseases, including ischemic stroke [13]. However, although observational studies strongly suggest an inverse association between vitamin D levels and CV risk and disease and potential mechanisms by which vitamin D may act (e.g., oxidative stress and inflammation), randomized controlled trials do not conclusively demonstrate a protective role of vitamin D supplementation on CV risk [4].

Interestingly, recent advances in vitamin D biology raised the complexity of this scenario by providing mechanistic alternatives to the different, and sometimes opposite, effects of vitamin D. Indeed, the identification of new, alternative pathways of vitamin D activation led to the discovery of additional biologically active metabolites that may interact not only with VDRs but also with other nuclear receptors, opening up a new area of research that requires further investigation to better understand the pleiotropic effects of vitamin D [14]. In particular, some reports have shown that, like 1,25(OH)D, vitamin D3 also relies on the direct binding to VDR to exert its effects [15,16].

Sphingolipids (SLs) are structural elements of the cellular membrane and key components of ceramides (Cer), a group of bioactive molecules characterized by the presence of sphingoid bases in the hydrophobic region of their molecular structure, while phosphate groups, sugar residues, and/or hydroxyl groups build up the hydrophilic region. Several studies attribute to Cer a wide range of functions, thus linking them to several important physiological pathways (e.g., apoptosis, cell proliferation, enzymatic activities, inflammation) based on their specific structure (e.g., fatty acyl chain length and subcellular location) [17]. Many studies also highlighted a connection between SLs, Cer, and cardiometabolic conditions, especially when considering the pathogenesis and evolution of type 2 diabetes, as well as acute and chronic coronary artery disease [18]. Moreover, numerous experimental and clinical findings demonstrate the key role played by SLs in CV pathophysiology, particularly in ischemic heart failure and disease, hypertension, acute myocardial infarction, and stroke [19]. In particular, as reported by previous works, specific ceramide species are associated with CV risk, inflammation, and disease severity in AMI [20,21].

Interestingly, a growing body of scientific evidence suggests that targeting SLs may benefit inflammation and cardiometabolic diseases, thus representing a promising and novel therapeutic option to be developed and investigated [22]. In this context, a study on SL levels in homogenates from 200 human carotid plaques demonstrated their association with inflammation and plaque instability, thus suggesting SLs as possible biomarkers of plaque vulnerability and potential pharmacological targets [23].

However, despite the encouraging results available, there are still challenges and limitations to overcome (e.g., standardization procedures, definition of reference values, and biological variability) before SLs can be introduced in clinical practice as biomarkers for diagnosis, management, and therapeutic targets for CM diseases [18,24,25].

Due to the high complexity of this class of molecules, also taking into account their multiple and sometimes opposite biological functions, SL pathophysiology definitely represents a scientific challenge; however, current knowledge needs to be further deepened in order to be able to target specific sphingolipids without affecting other species, thus eliciting collateral adverse effects [26].

Given the importance of vitamin D and SLs in cardiometabolic settings, it is worth highlighting that vitamin D, alongside many other actions (e.g., effects on endothelial function and smooth muscle cell activity, renin–angiotensin–aldosterone system, nitric oxide levels, oxidative stress, and inflammatory responses), is also involved in SL metabolism regulation. In addition, Cer act as mediators of 1,25(OH)D on cell differentiation, thereby playing a significant role in the mechanism of action of 1,25(OH)D, suggesting a crosstalk between these molecular entities and common effectors (e.g., growth factors, oxidative stress, and inflammation) [27,28,29].

Hence, this review aims to outline vitamin D metabolism and discuss the link between vitamin D and SLs in preclinical and clinical investigations, as well as current pitfalls and potential future opportunities in this field. Moreover, considering that recent scientific results have shown that vitamin D3 can bind directly to VDRs with a much higher affinity than 1,25(OH)D when bound to vitamin-D-binding protein (VDBP) [15,16], this review mainly aims to discuss the current available data on the interaction between sphingomyelin and 1,25(OH)D, without excluding the possibility of an interaction with 25(OH)D, which remains open for further investigations.

## 2. Vitamin D Biosynthesis and Metabolism

**Figure 1:**
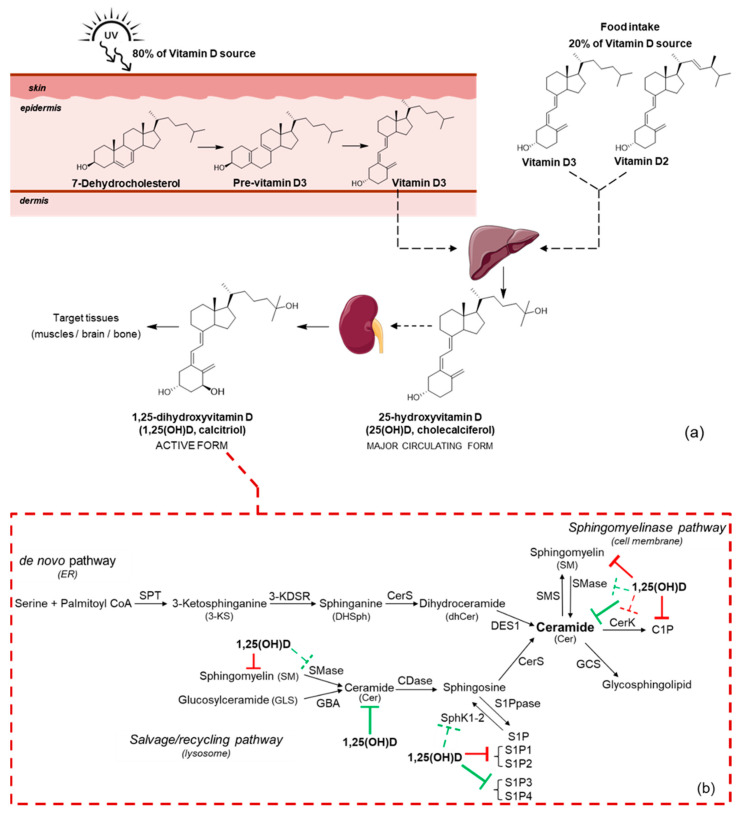
Vitamin D synthesis and metabolism (panel **a**); 1,25(OH)D, mainly derived from vitamin D3, and sphingolipid putative interactions (panel **b**): green lines denote activation/induction; red lines inhibition/blocking (dashed lines: weaker evidence).

From a biochemical standpoint, vitamin D is a biologically inactive prohormone that needs hydroxylation reactions to exert its properties efficiently. This fat-soluble secosteroid exists in two main forms, vitamin D2 (ergocalciferol, vitamin D2) and vitamin D3 (cholecalciferol, vitamin D3), chemically different in the side chain; indeed, unlike vitamin D3, vitamin D2 carries a double bond between C22 and C23 and a methyl group at C24 (Figure 1) [30,31,32]. These structural differences determine a different catabolism: indeed, vitamin D2 has a lower affinity for VDBP, so it is removed from the bloodstream more easily and quickly [30]. Although the main source of vitamin D in the body is represented by vitamin D3, both vitamin D2 and vitamin D3, despite their differences, contribute to human health and are available as vitamin supplements. A daily healthy diet provides vitamin D2 mainly from the plant sterol ergosterol through UV-B irradiation of the ergosterol, while vitamin D3 derives especially from animal foods like fish and eggs; however, as in the text we essentially refer to vitamin D3 (due to its amount in the human body), both forms are then metabolized in the same way and are referred to as vitamin D.

Nevertheless, dietary foods provide only a small percentage of the body’s vitamin D requirements, while the skin, particularly when exposed to sunlight, is the main vitamin D3 source, providing at least 80% of the vitamin D demand in humans [33,34]. In the epidermis, nonenzymatic photolysis, carried out by UV-B rays in the range of 280–320 nm, converts 7-dehydrocholesterol (7DHC, provitamin D3), an intermediate in cholesterol synthesis, into previtamin D3 (PreD3) through a rearrangement of the 7DHC B-ring in C9–C10 positions (Figure 1). Thereafter, a thermal isomerization of PreD3 to vitamin D3 takes place through the shift of a hydrogen from C19 to C9, completing the formation of vitamin D3. This last step is a rather slow, thermosensitive, and reversible reaction so that both PreD3 and vitamin D3 coexist (Figure 1) [30,31,32,35,36]. Hence, vitamin D3 biosynthesis relies mainly on the bioavailability of 7DHC and, as a consequence, on the activity of the enzyme 7-dehydrocholesterol reductase (DHCR7), which in the skin reduces 7DHC into cholesterol, according to the Kandutsch/Russel pathway [31,32].

Upon prolonged exposure to UV or solar radiation, a physiological control mechanism kicks in, and through the inhibition of vitamin D synthesis, PreD3 is converted to inactive metabolites (e.g., lumisterol and tachysterol) rather than vitamin D3. In this way, negative effects arising from excessive vitamin D synthesis are avoided [30].

### 2.1. Biosynthesis of 25-Hydroxyvitamin D in Liver

Once produced in the skin, vitamin D3 is released into the circulation and transported in serum through binding to VDBP. As a fat-soluble vitamin, a percentage of the newly synthesized vitamin D is also stored in the adipose tissue. Right after its synthesis, vitamin D has no biological activity but rather needs to be metabolized through two subsequent hydroxylation reactions, which lead to the active hormone 1,25(OH)D [30].

The first reaction is a 25-hydroxylation, which takes place mainly in the liver: here, in the microsomal P450 fraction of hepatocytes, the enzyme CYP2R1 25-hydroxylates both vitamin D2 and vitamin D3, yielding 25-hydroxyvitamin D (25(OH)D). Although CYP2R1 is considered the major enzyme responsible for this conversion, other enzymes have shown the same activity, thus contributing to the levels of 25(OH)D [31,35]. Among these, CYP27A1 is located mainly in liver and skeletal muscle tissue mitochondria and is also involved in bile acid synthesis [30]. However, the metabolism of vitamin D2 and vitamin D3 differ in some aspects: indeed, vitamin D3 is hydroxylated at positions 25- and 27- by CYP27A1, whereas vitamin D2 is hydroxylated at positions 24- and 27- by CYP27A1 [37].

### 2.2. Production of Active Vitamin D (1,25 (OH)D) in the Kidney

25(OH)D is the major circulating form of vitamin D and is, therefore, considered the main parameter to look at in order to obtain the best estimation of the body’s vitamin D pool. However, a second reaction is needed to obtain active vitamin D, namely 1,25(OH)D, with paracrine and autocrine functions. This second step is catalyzed by the enzyme 1-alpha-hydroxylase CYP27B1, mainly expressed in renal cells and, in a lower amount, in several other tissues, including keratinocytes, bone, placenta, and immune cells [32,35,36]. CYP27B1 hydroxylates the first carbon in the A ring of 25(OH)D, thus forming 1,25(OH)D.

The known half-life of 25(OH)D is around 15–20 days, and most of the circulating vitamin D is transported by VDBP (85%) and albumin (15%) to target tissues (e.g., kidney, muscle, brain, adipose tissue, bone), where it exerts its physiological function [30,32].

## 3. Sphingolipid Synthesis and Metabolism

SLs are synthesized via three main pathways, briefly discussed below:

De novo pathway: This pathway, which takes place in the cytosolic leaflet of the endoplasmic reticulum and its associated membranes, leads to the formation of Cer and sphingosine 1-phosphate (S1P) mainly through four reactions. In the first reaction, serine palmitoyltransferase (SPT) condenses palmitoyl-CoA and serine into 3-ketosphinganine (3-KS); then, through a second reaction carried on by a 3-keto-dihydrosphingosine reductase (3-KDSR), 3-KS is converted into sphinganine (DHSph), which is then further acetylated to dihydroceramide (dhCer) through different isoforms of ceramide synthase (CerS). CerS indeed promotes the reaction between acyl-CoA (made up of different chain lengths) and the molecule of sphinganine. Finally, a dihydroceramide desaturase introduces a *trans*-double bond in position C4–C5 trans of dhCer, thus forming the correspondent ceramide (Figure 1) [24,26].

Sphingomyelinase pathway: In this pathway, the hydrolysis of sphingomyelin (SM), carried out by sphingomyelinases (SMases), leads to the production of ceramides and phosphocholine. SMases are enzymes differently expressed in the various tissues according to their pH and structural characteristics.

Salvage pathway: It takes place in acidic subcellular compartments, like endosomes and lysosomes. Through the SMases enzyme, SM is metabolized to sphingosine (Sph), which is then recycled to produce Cer through the action of CerS [24,26].

## 4. Vitamin D and Sphingolipids Crosstalk: Preclinical Evidence

Many studies have already reported vitamin D effects on SL metabolism and signaling, while others have demonstrated the role of SL on vitamin D levels and function, thus strongly suggesting a crosstalk between these pathways (Figure 1).

The first evidence that 1,25(OH)D modulates phospholipid metabolism at the intestinal, renal, bone, and parathyroid levels dates back to the 1980s [38]. Accordingly, a 1,25(OH)D-related increase in Cer levels by SM hydrolysis, which in turn affects cellular growth and differentiation, was reported more than 30 years ago in leukemic HL60 cells [39]. More recently, studies demonstrated that 1,25(OH)D causes a significant reduction in ceramide kinase (CerK), the enzyme responsible for the synthesis, expression, and content of ceramide-1-phosphate (C1P), as well as an increase in Cer levels, resulting in an antiproliferative effect in neuroblastoma cells [40]. Moreover, in circulating osteoclast precursor monocytes, 1,25(OH)D administration reduces S1P receptor 2 (S1P_2_) gene expression levels, a receptor that negatively regulates S1P [41]. Indeed, when sphingosine kinase 1 (SPHK1), sphingosine kinase 2 (SPHK2), and S1P receptor 1–5 (S1P_1–5_) mRNA expression levels were measured in monocytes isolated from healthy controls and type 2 diabetes patients, mRNA levels of S1P_1_ and S1P_2_ (linked to inflammation and insulin resistance) were downregulated by calcitriol in monocytes from healthy subjects as well as type 2 diabetes patients, while S1P_3_ and S1P_4_ gene expression (both involved in the synthesis of cytokines) was induced; moreover, while SPHK1 was upregulated by calcitriol in both groups, SPHK2 gene expression only increased in type 2 diabetes subjects [42].

In contrast to these data, a reduction in S1P_3_ gene expression has been reported in human breast cancer cells following calcitriol administration, suggesting cell-type-specific responses [43]. On the other hand, increased SPHK1 gene expression after calcitriol treatment was previously observed in in vitro studies performed by using both HL-60 (myeloid cell line) and human keratinocytes; specifically, in HL-60 cells, 1,25(OH)D enhanced the production of S1P, which may protect against ceramide-induced apoptosis, despite the increase in Cer levels, whereas, in keratinocytes, hydrolysis of SM has also been observed following vitamin D administration [27,44,45]. In addition, primary human melanocytes were protected from apoptosis thanks to a 1,25(OH)D-related increase in S1P production [46]. Other data have also shown that the calcitriol-induced protection of human fibroblasts from apoptosis is actually due to an increase in S1P production, resulting in a change in the Bcl-2/Bax ratio (i.e., an increased Bcl-2 level while the Bax protein expression does not change) [47].

In the cardiometabolic (CM) field, recent data reported that vitamin D supplementation improves left ventricular dysfunction in a mouse model of diet-induced type 2 diabetes (10 weeks); at the 25th week, unsupplemented mice had increased myocardial levels of two lipotoxic species, Cer and diacylglycerol (DAG), which correlated with adverse effects at the cardiomyocyte level (e.g., ventricular hypertrophy, mitochondrial dysfunction, altered insulin signaling), which were normalized by vitamin D3 supplementation [48]. As previously reported, treatment with 1,25(OH)D can affect S1P receptor and SPHK expression, as well as S1P levels, in primary monocytes of both healthy controls and patients with type 2 diabetes [42].

Interestingly, vitamin D affects the lipid composition (including SL) of adipocyte-derived extracellular vesicles, playing key roles in energy homeostasis [49]. 1,25(OH)D boosts TNF-a expression (apoptosis inducer), which raises ceramide (apoptosis inducer) levels via SM hydrolysis [28]. However, concentration-dependent mechanisms are involved in vitamin D effects; indeed, in human keratinocytes, physiological levels of 1,25(OH)D did not enhance apoptosis despite increased expression of TNF-a and Cer [44]. This cytoprotective effect seems to be linked to S1P production since it was completely abolished by the N, N-dimethylsphingosine (a sphingosine kinase -SK inhibitor that blocked S1P generation); conversely, S1P may restore the beneficial effect of vitamin D despite the N, N-dimethylsphingosine presence [44].

Recent data obtained in HN9.10e cells showed that vitamin D may modulate cell differentiation by changing exosome lipid composition (SM decrease and consequent Cer accumulation, which may cause cell differentiation) [50]. Moreover, if VDR is considered a critical factor for epidermis-specific SL production and barrier formation, in favor of the existence of a crosstalk, changes in the composition of SLs in lipid rafts (lipid-rich microdomains) might influence the localization and functioning of vitamin D receptors [17,51]. Accordingly, SMase treatment alters the composition of nuclear microdomains by reducing 1,25(OH)D receptors in embryonic hippocampal cells (HN9.10e cell line), thus affecting 1,25(OH)D-induced cell differentiation [52]. Moreover, it has been observed that some ceramide derivatives may increase 1,25(OH)D-induced differentiation through PI3-K/PKC/JNK/ERK pathways in HL-60 cells [53]. In addition, a shift in SM composition (from C24:0-SM to C16:0-SM) has been associated with a lower expression of vitamin D receptors in cancer cells [54]. In general, S1P improves growth and survival, whereas its precursors (e.g., ceramide) induce growth arrest and cell death [55]. Nonetheless, this scenario is further complicated by the fact that ceramides with different molecular structures (different acyl chain compositions) hold different functions [17]. Additionally, some SLs might act as beneficial, while other species may contribute to lipotoxicity and damage. In this context, compensatory mechanisms can occur following SL changes that may alter ceramide homeostasis. For example, CerS2 (ceramide synthase 2), one of the six isoforms of CerS, induces the synthesis of very-long-chain ceramides, such as C24, C24:1. However, in CerS2-downregulated cells, the increase in the expression of CERS5 and CERS6, responsible for the synthesis of long-chain ceramides (C14- and C16), leads to a reduction in both C24-ceramide and SM, but also to an increase in C14- and C16-ceramide levels, with an alteration in overall SL metabolism and cellular homeostasis [56,57]. SLs may also have different effects on different cellular types, as SM stimulates vitamin-D-induced HL-60 cell differentiation while inhibiting vitamin-D-induced keratinocyte differentiation [57].

Furthermore, it should be considered that SL, in particular S1P, and 1,25(OH)D, can share common effectors (e.g., calcium regulation, growth factor expression, cytokine production, insulin, and glycemia levels), while their synergistic effects are still unclear and deserve further investigations.

Interestingly, studies showed that lysosomal ceramides, as well as vitamin D administration, modulate medial calcification in the arterial wall of smooth-muscle-cell-specific Smpd1 transgenic mice, suggesting that these factors might interact in the changes that contribute to the development of medial arterial calcification and arterial stiffness [58].

## 5. Vitamin D and Sphingolipids Crosstalk: Clinical Evidence

Only a few human studies have assessed the association between vitamin D deficiency and its interaction with SL metabolism and signaling in metabolic and cardiometabolic diseases. In particular, vitamin D deficiency was associated with several metabolic complications and diseases, including hypertension, obesity, insulin resistance, and diabetes [59,60]. It is also well known that insulin resistance is linked to SL metabolism: specifically, ceramides play a role in downstream insulin receptor signaling pathways, e.g., phosphorylation of insulin receptor substrate 1, activation of Akt/PKB and phosphatidylinositol 3-kinase, blocking the translocation of glucose transporter GLUT4, and apoptosis of pancreatic β-cell [61,62].

In patients with type 2 diabetes, plasma levels of Cer, Sph, DHSph, and their phosphate derivatives were measured at the beginning of the study, after 6 months of vitamin D supplementation, and after another 6 months of follow-up. Results showed that both at baseline and 6 months after follow-up, there were no differences in plasma SL species between placebo and patients. However, after 6 months of supplementation, vitamin D significantly enhanced plasma levels of C18 dhCer (N-stearoyl-sphinganine (d18:0/18:0)) and C18 Cer (N-stearoyl-sphingosine (d18:1/18:0)) in patients with type 2 diabetes compared with the placebo group. Other dhCer and Cer remained, instead, unchanged after 6 months of vitamin D supplementation, as unchanged were also the plasma levels of sphingosine, DHSph, and their phosphate derivatives (S1P and DhS1P) [63]. These results are in agreement with a study by Wigger et al., in which they demonstrated, up to 9 years before the onset of type 2 diabetes, an increase in dhCer levels in individuals who later developed the disease, thus showing that these lipids may potentially serve as early biomarkers for the development of type 2 diabetes [64]. The biological effect of ceramides depends on the chain length of the fatty acid linked to the sphingosine backbone: N-palmitoyl-sphingosine (Cer d18:1/16:0), for example, is the principal mediator of obesity-related insulin resistance, while N-stearoyl-sphingosine (Cer d18:1/18:0, C18 Cer) is associated with insulin resistance [65,66].

The role of vitamin D supplementation in 70 overweight/obese African Americans was recently investigated in order to determine whether its supplementation altered circulating long-chain ceramides and their metabolites. For 16 weeks, subjects were randomly assigned to one of four groups: 600, 2000, or 4000 IU/day vitamin D3 supplements or placebo. In this study, serum levels of N-stearoyl-sphingosine (Cer d18:1/18:0) and stearoyl sphingomyelin (Cer d18:1/18:0) significantly increased after vitamin D3 supplementation in a dose–response manner, while other metabolites such as N-palmitoyl-sphingosine (Cer d18:1/16:0), N-palmitoyl-sphinganine (Cer d18:0/16:0), Sph, S1P and Cer d18:1/16:0 remained unchanged. Moreover, changes in N-stearoyl-sphinganine (Cer d18:0/18:0), Sph, and S1P were positively associated with changes in hemoglobin A1c, while Cer d18:1/18:0 and Cer d18:0/18:0 were associated with changes in BMI [67].

A proteomic study on vitamin-D-deficient Saudi Arabian subjects identified several proteins (belonging to different pathways, i.e., lipid metabolism, vitamin D function, and immunity/inflammation) that are differentially expressed in obese versus lean people [68]. Using mass spectrometry on the sera of these same subjects, it was possible to identify specific peptides (involved in lipid metabolism and inflammation processes) that are associated with obesity and vitamin D deficiency [69]. In line with these findings, the pattern of SL was studied in three categories of Saudi men and women, namely normolipidemic with normal weight (NW), vitamin-D-deficient subjects, and dyslipidemic subjects, who were either obese (-vitamin DO) or had normal BMI (-vitamin DNW), with the aim to investigate SL changes in dyslipidemia and vitamin D deficiency, as well as their contribution to obesity. Overall, this study showed an increase in total ceramides in dyslipidemic (both -vitamin DNW and -vitamin DO) compared with normolipidemic NW subjects, suggesting a link between total Cer abundance with dyslipidemia and vitamin D deficiency. Furthermore, this same study also highlighted that the increase in total Cer levels is independent of obesity since they were significantly higher not only in obese but also in dyslipidemic normal-weight subjects. However, total SM and dihydrosphingomyelins (dhSM) were lower in both -vitamin DNW and -vitamin DO than NW, likely due to the direct correlation between this class of molecules and vitamin D deficiency and hyperlipidemia [70]. Moreover, men with obesity had lower levels of specific acyl chains of Cer (Cer C20:0 and C24:2) and higher levels of dhSM C18:0, while women with obesity had lower concentrations of Cer C24:1 and higher levels of SM C16:0 [70].

In another cross-sectional, retrospective study, based on data from 277 participants randomly selected from a larger cohort of 1820 subjects, lipidomic profiling was investigated in relation to vitamin D status and dyslipidemia. This analysis highlighted, on the one hand, higher SM levels in subjects with both physiological levels of vitamin D and dyslipidemia, while in vitamin-D-deficient patients either with or without dyslipidemia, phosphatidylcholine (PC) and phosphatidylethanolamine (PE) were highly altered. Furthermore, levels of SL and ceramide-related metabolites in normo- and dyslipidemic subjects with vitamin sufficiency and deficiency were examined in the same study, revealing higher levels of N-stearoyl-sphingosine (Cer d18:1/18:0) in dyslipidemic and vitamin-D-deficient subjects [71].

Taken together, these results show a significant alteration of the metabolomic profiles in dyslipidemic and vitamin-D-deficient individuals when compared with control groups, an alteration that reflects changes in key physiological pathways and that involves several classes of biological functional metabolites, including Cer, lysophospholipids, phosphatidylcholines, phosphatidylethanol amines, and SM.

A more recent study investigated sphingolipid levels in the red blood cell membrane, as well as their relationship with some well-established biomarkers of cardiovascular health, including vitamin D status in healthy women (30–60 years; mean age 46.2 ± 8.0) [72,73]. The results obtained from this study showed a direct correlation between serum vitamin D status and erythrocytes’ sphingolipid levels, suggesting not only that vitamin D levels are related to sphingolipid erythrocyte content but also that this association may have repercussions for CM risk in view of the role that vitamin D, as well as SLs, have for CM pathophysiology. Prior to these results, only a few studies analyzed phospholipid fractions and sphingolipid content in erythrocyte membranes, which, however, have the advantage of reflecting the lipid profile over a long period of time. Interestingly, changes in the phospholipid composition of the erythrocyte membrane have been observed in Alzheimer’s and Parkinson’s disease, but also in coronary artery disease, thus pointing to shared pathological mechanisms underlying these chronic diseases, as well as the possibility for erythrocyte membrane sphingolipid content to act as biomarkers for these conditions [74].

The crosstalk between vitamin D and SLs has also been hypothesized for the nervous system [75]. As in the CM field, it is well known that vitamin D and SLs share common targets (e.g., calcium metabolism, growth factor expression, and levels of inflammatory biomarkers) in this physiological district; however, it is not entirely clear yet whether vitamin D and SLs cooperate to induce a final effect or act separately through common effectors [75]. Interestingly, a Parkinson’s disease experimental model suggested that the vitamin-D-related stimulation of neutral sphingomyelinase activity reduces specifically saturated fatty acid SM, thus affecting neurite plasticity [76]. Moreover, in order to evaluate whether SLs are involved in the protective action of vitamin D, Pierucci et al. demonstrated how 1,25(OH)D and its structural analog ZK191784 are able to oppose the Aβ(1–42) peptide-induced toxicity through modulation of the S1P/S1P1/p38MAPK/ATF4 pathway in differentiated human neuronal SH-SY5Y cells, thus modulating the balance between neurodegeneration and neuroprotection [77].

However, although changes in the vitamin D and SL signaling pathways, as well as in blood levels, have been observed in patients with neurodegenerative diseases, few studies have actually demonstrated a crosstalk between vitamin D and SLs [75]. Interestingly, in their study, Zhu et al. evaluated the relationship between vitamin D and circulating and intracellular levels of gelsolin, a regulator of S1P, which is downregulated in patients with multiple sclerosis. Their results showed that vitamin D may reduce S1P and inflammation but also that these beneficial effects are limited by a reduction in gelsolin-related effects [78]. These findings clearly highlight how little is known about the effects of vitamin D on SLs and vice versa, as well as the complexity of their connection, which is further exacerbated by compensatory events and the activation of additional and synergistic cellular pathways that might also contribute to adverse side effects.

## 6. Conclusions

Vitamin D affects multiple biological systems, playing a key role in the prevention and regulation of cardiometabolic diseases (e.g., modulating levels of inflammatory and oxidative mediators, controlling cellular proliferation and growth, insulin resistance, obesity, and type 2 diabetes), which are largely the same effectors targeted by SLs. Not surprisingly, available data clearly support the existence of a crosstalk between the SL and vitamin D pathways (Figure 1): indeed, this interaction seems to potentially drive the beneficial effects reported with the supplementation of cholecalciferol and its analogs. However, the clinical implications of this relationship remain complex and require further evaluation and more detailed mechanistic studies. Likewise, the characterization of further biological properties associated with other molecules in the vitamin D pathway beyond 1,25(OH)D and an in-depth study of their roles in SL metabolism also deserve further investigation, as does a precise and accurate identification and elicitation of those SLs that can act as reliable biomarkers. In this context, the option to combine vitamin D supplementation with SL-targeted drugs may have clinical relevance and should be tested in the future in order to gain a better understanding and deep knowledge of the onset and development of cardiometabolic alterations, as well as the ability to prevent complications and adverse events in this widely spread and still challenging clinical field [79].

## Data Availability

Not applicable.

## Abbreviations

1,25(OH)D	1,25-dihydroxyvitamin D; calcitriol
VDR	Vitamin D receptor
RXR	9-*cis*-retinoic acid receptor
VDRE	Vitamin D response element
mVDR	Membrane-VDR
MARRS	Membrane-associated rapid response steroid-binding protein
CV	Cardiovascular
SL	Sphingolipid
Cer	Ceramides
VDBP	Vitamin-D-binding protein
7DHC	7-dehydrocholesterol/provitamin D3
PreD3	Previtamin D3
DHCR7	7-dehydrocholesterol reductase
25(OH)D	25-hydroxyvitamin D
S1P	Sphingosine-1-phosphate
SPT	Serine palmitoyltransferase
3-KS	3-ketosphinganine
3-KDSR	3-keto-dihydrosphingosine reductase
DHSph	Sphinganine
CerS	Ceramide synthase
dhCer	Dihydroceramide
SM	Sphingomyelin
SMases	Sphingomyelinases
CerK	Ceramide kinase
C1P	Ceramide-1-phosphate
S1P_1–5_	S1P receptors 1–5
SPHK1–2	Sphingosine kinases 1 and 2
CM	Cardiometabolic
DAG	Diacylglycerol
SK	Sphingosine kinase
PI3-K	Phosphoinositide 3-kinase
PKC	Protein kinase C
JNK	c-Jun N-terminal kinase
ERK	Extracellular signal-regulated kinase
NW	Normolipidemic with normal weight
vitamin DO	Vitamin-D-deficient dyslipidemic obese (subjects)
vitamin DNW	Vitamin-D-deficient dyslipidemic normal weight (subjects)
dhSM	Dihydrosphingomyelins
